# M867, a Novel Selective Inhibitor of Caspase-3 Enhances Cell Death and Extends Tumor Growth Delay in Irradiated Lung Cancer Models

**DOI:** 10.1371/journal.pone.0002275

**Published:** 2008-05-28

**Authors:** Kwang Woon Kim, Luigi Moretti, Bo Lu

**Affiliations:** Department of Radiation Oncology, Vanderbilt Ingram Cancer Center, Vanderbilt University School of Medicine, Nashville, Tennessee, United States of America; National Institutes of Health, United States of America

## Abstract

**Background:**

Lung cancer remains the leading cause of cancer death worldwide. Radioresistance of lung cancer cells results in unacceptable rate of loco-regional failure. Although radiation is known to induce apoptosis, our recent study showed that knockdown of pro-apoptotic proteins Bak and Bax resulted in an increase in autophagic cell death and lung cancer radiosensitivity *in vitro*. To further explore the potential of apoptosis inhibition as a way to sensitize lung cancer for therapy, we tested M867, a novel chemical and reversible caspase-3 inhibitor, in combination with ionizing radiation *in vivo* and *in vitro*.

**Methods and Findings:**

M867 reduced clonogenic survival in H460 lung cancer cells (DER = 1.27, p = 0.007) compared to the vehicle-treated treated cells. We found that administration of M867 with ionizing radiation in an *in vivo* mouse hind limb lung cancer model was well tolerated, and produced a significant tumor growth delay compared to radiation alone. A dramatic decrease in tumor vasculature was observed with M867 and radiation using von Willebrand factor staining. In addition, Ki67 index showed >5-fold reduction of tumor proliferation in the combination therapy group, despite the reduced levels of apoptosis observed with terminal deoxynucleotidyl transferase-mediated dUTP nick end labeling staining. Radiosensitizing effect of M867 through inhibiting caspases was validated *using* caspase-3/-7 double-knockout (DKO) mouse embryonic fibroblasts (MEF) cell model. Consistent with our previous study, autophagy contributed to the mechanism of increased cell death, following inhibition of apoptosis. In addition, matrigel assay showed a decrease in *in vitro* endothelial tubule formation during the M867/radiation combination treatment.

**Conclusions:**

M867 enhances the cytotoxic effects of radiation on lung cancer and its vasculature both *in vitro* and *in vivo*. M867 has the potential to prolong tumor growth delay by inhibiting tumor proliferation. Clinical trials are needed to determine the potential of this combination therapy in patients with locally advanced lung cancer.

## Introduction

Lung cancer is the most prevalent cancer worldwide, and the leading cause of cancer-related mortality despite multi-modality therapy, resulting in 160,390 estimated deaths in the United States only in 2007 [1]. Since tumor resistance limits effectiveness of current treatments [2], [3], [4], such as radiotherapy, the identification of novel therapeutic strategies is critical for improving outcome. Of the different cell death processes involved in cancer treatment, apoptosis has been the most well-studied [5]. During apoptosis, the major mediators of cellular destruction are effectors caspase-3 and -7, members of the cysteine aspartate proteases family, which cleave proteins after an aspartate residue [6]. Although radiation is known to induce apoptosis, yet it accounts for a minor portion of cell death in irradiated solid tumors [7], and our recent study showed that knockdown of pro-apoptotic proteins Bak and Bax resulted in an increase in lung cancer radiosensitivity *in vitro* [8]. Autophagy, a non-apoptotic cell death type, was shown to contribute to the mechanism of increased cell death.

Autophagy is an evolutionarily conserved process and a survival mechanism allowing for the recycling of long-lived organelles and proteins [9], [10]. Autophagy also serves as a suicide pathway with complete self-digestion under excessive stress conditions [11], [12], which is classified as programmed cell death Type 2 [9], [10]. During autophagy, organelle degradation occurs through formation of cytoplasmic double-membrane vacuoles, called autophagosomes with intact nuclei [13]. Autophagy regulation has been of increasing interest, because of its implication in tumorigenesis. In addition, prolonged autophagy can lead to cancer cell death, leading to the hypothesis that autophagy can be exploited as a cancer therapy target [14], [15], [16], [17].

To further explore the potential of apoptosis inhibition as a way to sensitize lung cancer for therapy, we tested M867, a novel chemical and reversible caspase-3 inhibitor [18], in combination with ionizing radiation *in vivo* and *in vitro*.

## Materials and Methods

### Cell culture

Primary mouse embryonic fibroblasts (MEFs) were derived from wild-type (WT) and Caspase3^−/−^/7^−/−^ double knockout (DKO) mice and immortalized by transfection with a plasmid containing SV40-T-antigen (provided by Dr. Richard Flavell, Yale University, New Haven, CT). MEFs were cultured in DMEM (Invitrogen Corporation) supplemented with 10% fetal bovine (FBS), 1% penicillin-streptomycin and 0.5 μmol/L 2-mercaptoethanol. H460 lung cancer cells were cultured in RPMI 1640 (Invitrogen, Grand island, NY) supplemented with 10% fetal bovine serum and 1% penicillin-streptomycin at 37°C and humidified 5% CO2. HUVECs were obtained from Clonetics and were maintained in EBM-2 medium supplemented with EGM-2 MV single aliquots (BioWhittaker). M867 was obtained from Merck & Co., Inc.

### Clonogenic Assay

H460 lung cancer cells were treated with DMSO or M867 (1.4nM, 5nM, and 10nM for 24hrs); WT MEFs cells and caspase 3/7 DKO MEFs cells were treated with DMSO or M867 (5nM and 10nM for 24hrs); and MEFs cells were treated with siRNAs against caspase-3 and-7, siRNAs against Beclin-1 and ATG5, or control. Cells were then irradiated with 0 to 6 Gy as indicated at a dose rate of 1.8 Gy/min, by using of a ^137^ Cs irradiator (J.L. Shepherd and Asssociates, Glendale, CA). After irradiation, cells were incubated at 37°C for 8–10 days. Cells were fixed for 15min with 3:1 methanol: acetic acid and stained for 15min with 0.5% crystal violet (Sigma) in methanol. After staining, colonies were counted using a cutoff of 50 viable cells. Surviving fraction was calculated as (mean colony counts)/(cells inoculated)×(plating efficiency (PE)), where PE was defined as (mean colony counts)/(cells inoculated for non-irradiated controls). DER was calculated as the dose (Gy) for radiation alone divided by the dose (Gy) for radiation plus M867 (normalized for M867 toxicity) necessary for a surviving fraction of 0.25. Experiments were conducted in triplicate and mean, SD, and P values were calculated.

### Immunoblotting

Cells (0.5×10^6^) were treated with various Gy and drugs. They were collected at various time points, and then were washed with ice-cold PBS twice before the addition of lysis buffer (20mM Tris-HCl, pH 7.4, 150mM NaCl, 20mM EDTA, 1% NP40, 50mM NaF, 1mM Na3Vo4, 1mM NaMO4 and cocktail inhibitor (Sigma, 5ul/ml). Protein concentration was quantified by the Bio-Rad method. Equal amounts of protein were loaded into each well and separated by 12.5% SDS-PAGE gel, followed by transfer onto PVDF-membranes (BIO-RAD). Membranes were blocked by use 5% nonfat dry milk in PBS-T for 1h at room temperature. The blots were then incubated with Caspase-3 (Cell signaling); Caspase-7 (Cell signaling); LC-3 (Medical& Biological Laboratories Co. LTD); Akt, phospho-Akt (Ser-473), S6 ribosomal protein, and phospho-S6 ribosomal protein (Ser-240/244) (Cell Signaling); and Actin antibodies for 1hr at 4°C. Goat anti-rabbit IgG secondary (1:5000, Santa Cruse Biotechnologies) was incubated for 45min at room temperature. Immunoblots were developed by using the chemiluminescence detection system (PerkinElmer) according to the manufacture's protocol and autoradiography.

### Measurement of Apoptosis

Cells (2.5×10^5^) were plated into 10mm dishes for each data point. After 24 h of 37°C incubation, H460 cells were treated with M867 (100nM for 2 hours) and immediately irradiated with 5Gy, 10Gy or 20Gy, and WT and Caspase3^−/−^/7^−/−^ DKO cells were irradiated with 5Gy or 10Gy. After 24h, cells were treated with 1ml of Accutase (keeping all floating cells) for 4min and then counted for each sample. Cells were centrifuged and re-suspended in 1×Binding Buffer at a concentration of ∼1×10^6^ cells/ml. 100 μl of the solution (∼1×10^5^ cells) were transferred in 5ml FACS tube, added with 1.2 μl of Annexin V FITC and 1.2 μl of PI. After incubation for 30 min at RT in the dark, 400 μl of 1× Binding Buffer were added to each tube. The rate of apoptosis was measured using the Annexin V-fluorescein isothiocyanate apoptosis detection kit I (Pharmingen) with flow cytometry.

### Autophagy Assay

MEFs Cells were transfected with 2.5 μg of GFP-LC3 expression plasmid (gift from Dr. Norboru Mizushima) [19] using the lipofectamine reagent (Invitrogen Life Technologies). After 24h, cells were treated with 5Gy of radiation, with or without dose M867. After 24h and 48h, the fluorescence of GFP-LC3 was observed using confocal fluoroscopy.

### siRNA Transfection

siRNAs against mouse caspase-3 and-7, Beclin, and control siRNA were purchased from Santa Cruz Biotechnologies. siRNA ATG5(mouse) was synthesized by Dharmacon Research. The sense and antisense strands of ATG5 were begun at nucleotide, 5′-AACUUGCUUUACUCUCUCAUCAUU-3′ (Sense) and 3′-UUUUGAACGAAAUGAGAGAUAGU-5′. (Antisense) Cells were transfected with 25nM of siRNAs using LipofectAMINE 2000. The transfected cells were used for experiments 24h later.

### Endothelial Cell Morphorgenesis assay: Tubule Formation

HUVECs grown to ∼70% confluency were treated with 5nM M867, 3Gy, or combination therapy. Cells were then trypsinized and counted. They were seeded at 48000 per well on 24-well plates coated with 300 μl of Matrigel (BD Biosciences). These cells undergo differentiation into capillary-like tube structures periodically observed by microscopy. 24h later, cells were stained with hematoxylin and eosin (H&E) and photographs were taken via microscope. The average number of tubules was calculated from examination of three separate microscopic fields (100×) and representative photographs were taken.

### Tumor volume assessment

Human H460 lung cancer cells were used in a xenograft model in female athymic nude mice (nu/nu), 5–6 weeks old [Harlan Sprague Dawley Inc., Indianapolis, IN]). A suspension of 1×10^6^ cells in 50 μL volume was injected subcutaneously into the left posterior flank of mice using a 27½-gauge needle. Tumors were grown for 6–8d until average tumor volume reached 0.25cm^3^. Treatment groups consisted of vehicle control (in DMSO), M867, vehicle plus radiation, and M867 plus radiation. Each treatment group contained 5 mice. DMSO or M867 was given daily by intraperitoneal (i.p.) injection at doses of 2mg/kg for 7 consecutive days. In the case of combination treatment, drug or vehicle was given for 2d prior to the first dose of irradiation. Mice in radiation groups were irradiated 1h after drug or vehicle treatment with daily 2Gy fractions given over 5 consecutive days. Tumors on the flanks of the mice were irradiated using an X-ray irradiator (Therapax, Agfa NDT, Inc., Lewis Town, PA). The non-tumor bearing parts of the mice were shielded by lead blocks. Tumors were measured 2–3 times weekly in 3 perpendicular dimensions using a Vernier caliper and volume was calculated using the modified ellipse volume formula (volume = (height×width×depth)/2). Growth delay was calculated for treatment groups relative to control tumors.

### Histological sections, vWF, ki67 and TUNEL staining

Mice were implanted with H460 cells and treated as described above in the tumor volume studies. After 7d of daily treatments, mice were sacrificed and tumors were paraffin fixed. Slides from each treatment group were then stained for von Willebrand factor (vWF) using anti-vWF polyclonal antibody (Chemicon). Blood vessels were quantified by randomly selecting 400× fields and counting the number of blood vessels per field. This was done in triplicate and the average of the three counts was calculated. Ki67 and terminal deoxynucleotidyl transferase-mediated dUTP nick end labeling (TUNEL) staining were performed in the Vanderbilt University pathology core laboratory using standard protocols. Number of positive cells per field were scored and graphed by averaging three repeated assessments.

### Statistical analysis

Analysis of study results focused on testing the differences of the mean tumor volume among treatment groups and different time points. The data analysis was completed using the restricted/residual maximum likelihood-based mixed-effect model to adjust the intracorrelation effect for the mice that had multiple measurements. The model reported in the paper was selected on the basis of the Schwarz's Bayesian criterion. All tests of significance were 2-sided, and differences were considered statistically significant when p was less than 0.05. A statistical package, SAS v8.2, was used for all analyses.

## Results

### M867 inhibited apoptosis but enhanced radiation sensitivity in H460 lung cancer cells

**Figure 1:**
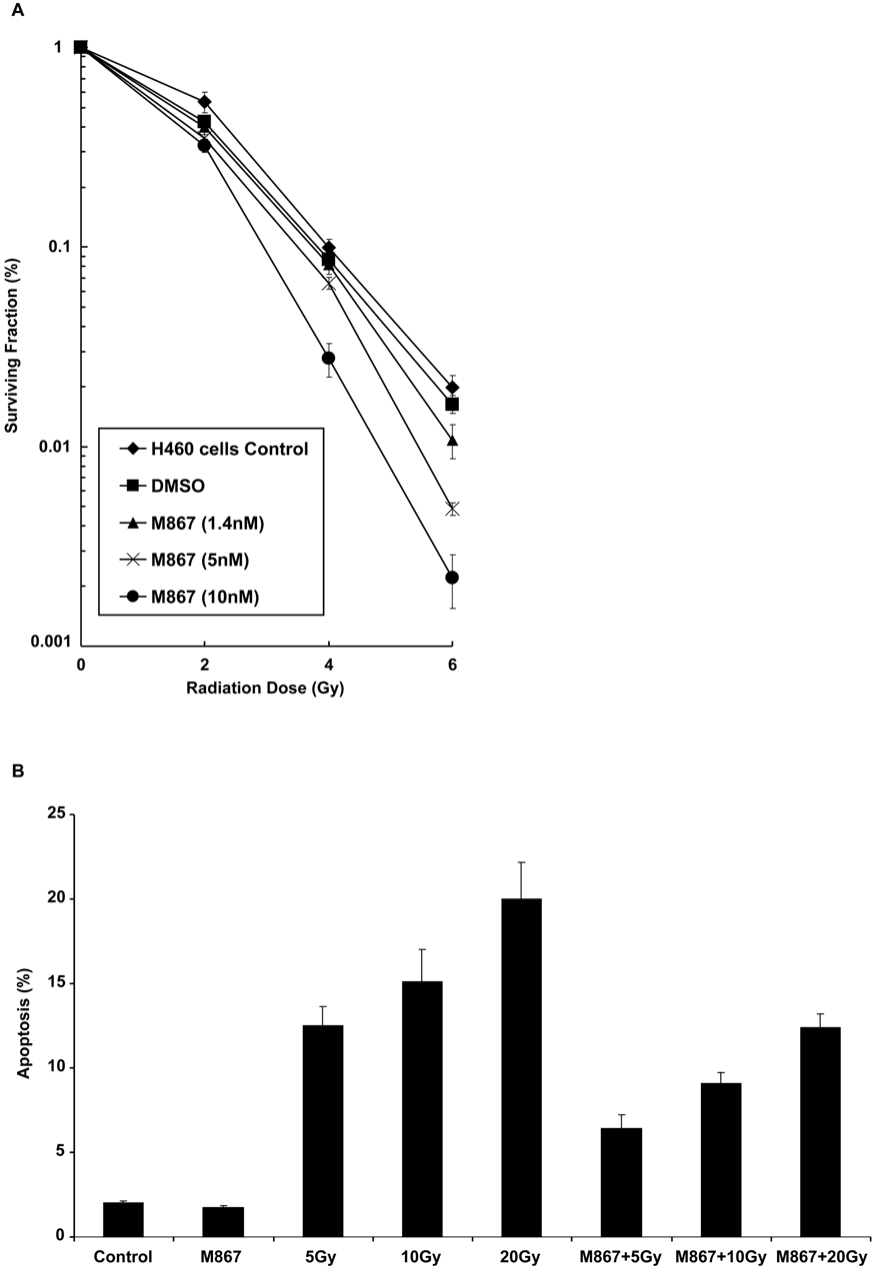
M867 inhibits apoptosis and reduces clonogenic survival of H460 lung cancer cells. (A) H460 NSCLC cells were treated with DMSO control, M867 (dose range from 1.4 to 10 nM, for 24hrs) with radiation. After 8 d, colonies were stained and the scored colonies were graphed. Survival curves for H460 cells±M867 treatment. Points, mean; bars, SD (*P* = 0.007). Shown are the mean +/− the standard deviation of three separate repeated experiments. (B) Annexin-V assay showing the level of apoptosis in H460 cells treated by either control, 100nM of M867 for 2h, radiation (5, 10 or 20 Gy), and both modality. This was done in triplicate and the average of the three counts was calculated. Columns, average; bars, SD.

To test the effect of inhibition of caspase-3 on lung cancer sensitivity to radiation, H460 cells were treated with novel reversible inhibitor M867 in combination with ionizing radiation. Surviving colonies were counted 8 days later. Clonogenic survival assay, shown in Figure 1A, demonstrated a decreased survival in various dose groups, with 10nM of M867 resulting in the greatest enhancement in radiosensitivity, as compared to control (DER = 1.27, p = 0.007). The level of apoptosis in H460 cells was measured after M867 treatment (100nM for 2h) and irradiation with 5Gy to 20Gy, using Annexin-V assay. It has been previously shown that Annexin V binding was blocked by M867 with an IC_50_ of 80 nM [20]. As shown in Figure 1B, the level of apoptosis was progressively elevated with increased doses of radiation. 20% of irradiated H460 cells were apoptotic at 20 Gy. When M867 was given prior to radiation, apoptotic cells were decreased to half at both low and high dose levels of radiation. These results demonstrate that sensitization of H460 lung cancer cells to ionizing radiation was associated with decreased apoptosis.

### Combined M867/radiation treatment prolongs tumor growth delay and is well-tolerated in lung xenograft model

**Figure 2:**
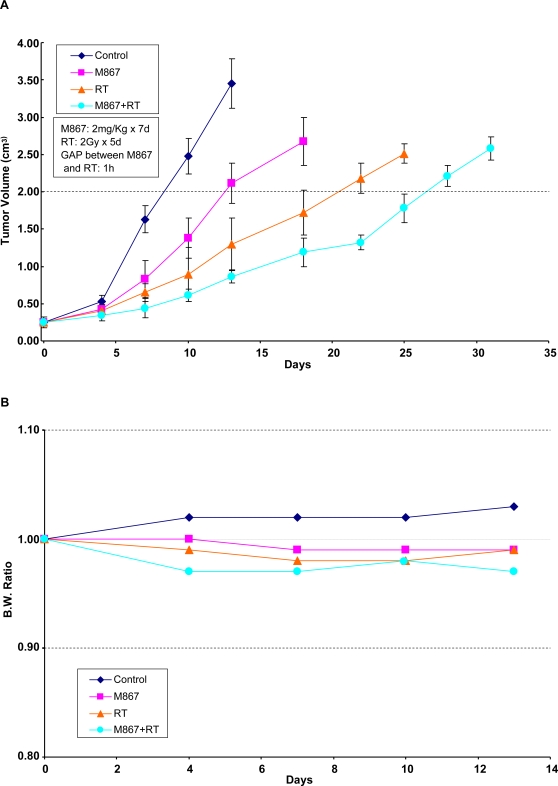
Extended tumor growth delay with M867 and radiotherapy at well-tolerated doses in xenografted H460 model. H460 lung cancer cells were xenografted subcutaneously in athymic mice. After 6–8 days, mice were treated daily for 7 days, with vehicle control, M867 (2mg/kg), and then were treated 1 hour after drug treatment with 2 Gy of radiation, daily over 5 consecutive days. Tumor was excised when reached the size of approximately 2.5–3.0 cm^3^ and tumor growth delay as defined by the number of days required to reach a tumor volume of 2 cm^3^ was measured. (A) Tumors were measured regularly and growth delay was calculated for treatment groups relative to control tumors. The combined bi-modality therapy induced a marked tumor growth delay when compared with radiation alone (26 vs 20 days, p<0.005). (B) Body weights were measured every 5 days and body weight ratio was calculated relative to baseline measurement.

Having established the effectiveness *in vitro* for M867-induced radiosensitization of lung cancer cells, a mouse hind limb xenograft model was generated to explore the radiation response by M867 *in vivo*. H460 lung cancer cells were injected subcutaneously into athymic nude mice, and tumors were allowed to grow for approximately 7 days to produce an average tumor volume of 0.25cm^3^ prior to therapy. The treatment groups consisted of a vehicle control, M867, vehicle plus radiation, and combination of M867 plus radiation. M867 was administered daily by 2mg/kg intraperitoneal injection for 7 consecutive days. Hind limb H460 tumor xenografts in mice were treated as described in the Material and Methods section. Growth delay was calculated as the number of days required to reach a tumor volume of 2 cm^3^ for treatment groups relative to control tumors. As shown in Figure 2A, a significant tumor growth delay was seen with combination therapy of M867 and radiation compared to irradiation alone (26 vs 20 days, p<0.005), and M867 alone did also significantly affect the tumor growth compared to control (∼4 days delay, p = 0.003). This suggests that M867 could increase tumor response in combination with radiotherapy. In addition, the body weight changes were also tracked in the mice to assess whether treatment with M867, radiation, or combination treatment yielded systemic toxicity (Figure 2B). Only minimal weight loss was seen at 10 days following the combined M867 and irradiation treatment. Changes in body weight after this time period reflected tumor growth. As expected, the combination treatment group, which had the most prolonged tumor growth delay, had the smallest increase in body weight.

### M867 reduces tumor proliferation index despite marked decrease in apoptosis in irradiated H460 mouse xenograft

**Figure 3:**
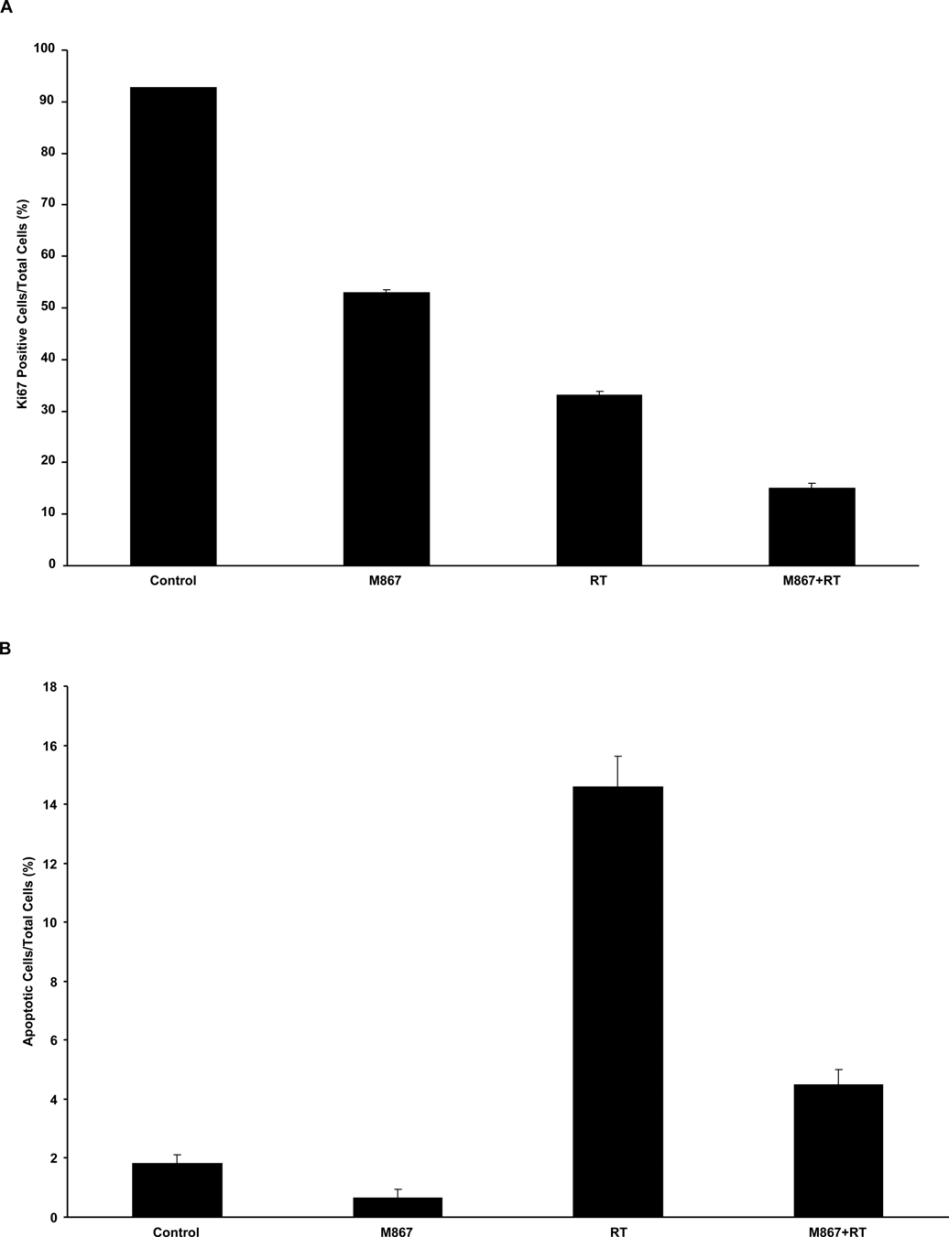
M867 with radiation reduces Ki67 proliferative marker and increases apoptotic index in H460 xenograft tumor models. Histologic sections were obtained from the tumors of the mice in each treatment group from the tumor volume study. Number of positive cells were scored and graphed by averaging three repeated assessments. (A) Average Ki67 proliferative index of each treatment group was determined by the percent of Ki67-positive cells per microscopic field. This was repeated thrice. Columns, mean; bars, SD. (B) TUNEL staining was also performed on tumor sections, and apoptotic index was similarly calculated by percent of positive TUNEL stained cells per microscopic field. Column, mean; bars, SD.

To determine the mechanism that contributes to the tumor growth delay following the combination therapy, Ki67 proliferative index was examined using fixed H460 tumor sections in all treatment groups. As shown in Figure 3A, M867 plus radiation combination treatment resulted in a 6-fold (15% vs 92%, p<0.001) and 2-fold (15% vs 33%, p<0.001) reduction in proliferating cells compared to control and radiation alone groups, respectively. We next assessed apoptosis levels in fixed H460 tumor sections using TUNEL staining. As shown in Figure 3B using TUNEL staining, combined M867 and radiation treatment resulted in two-thirds reduction in apoptosis (4.5% vs 15%, p<0.008), compared to radiation alone.

### M867 reduces vascular density in irradiated lung tumor model and sensitizes HUVECs to radiation

**Figure 4:**
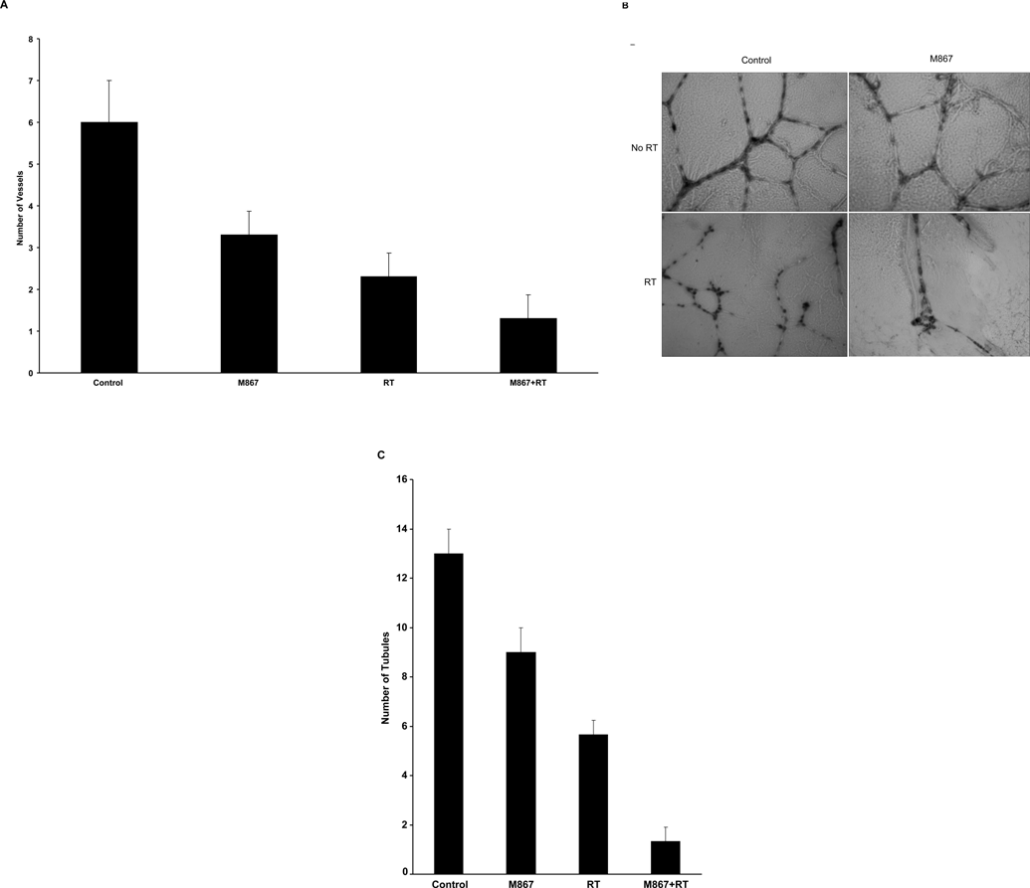
M867 sensitizes vascular endothelial *in vitro* model to ionizing radiation and reduces *in vivo* vascular density in irradiated H460 tumors. (A) Histologic sections were obtained from the tumors of the mice in each treatment group from the *in vivo* tumor volume study, and stained for blood vessels using an antibody for vWF. Blood vessels were quantified by randomly selecting 400×fields and counting the number of blood vessels per field. This was done in triplicate and the average of the three counts was calculated. Columns, average; bars, SD. (B) Human umbilical vein endothelial cells (HUVECs) were treated with 5 nM M867 and then immediately irradiated with either 0 or 3 Gy. Six hours later, cells were trypsinized and replated on 24-well plates coated with Matrigel. After 24 h, cells were fixed and stained with H&E. The slides were examined by microscopy (×100), and representative fields are shown. (C) Stained tubules were then counted in three separate, randomly selected fields. Columns, mean number of tubules counted per microscopic field; bars, SD.

Since tumor vasculature is a target for cancer treatment, mice were treated similarly to those in the tumor growth delay study and fixed lung tumor sections were used to determine the effects of M867 and radiation on tumor vasculature *in vivo*. Slides from each treatment group were analyzed following von Willebrand Factor (vWF) staining for vascular density study, as shown in Figure 4A. The number of vessels per microscopic field was then determined for each treatment group. Combination therapy of M867 and radiation (1.3; SD = 0.57) resulted in a dramatic 5-fold reduction in the average number of vessels per microscopic field in comparison to control (6; SD = 1; p<0.002) and a ∼2-fold reduction relative to radiation therapy alone (2.3; SD = 0.57; p<0.007) (Figure 4A).

To further investigate the effects of M867 and radiation on blood vessel formation, human umbilical vein endothelial cells (HUVECs) were used to examine tubules formation for angiogenic function *in vitro*. The endothelial cell morphogenesis assay was done to examine the ability of treated HUVECs to produce capillary-like tubular structures. A representative image and the mean number of counted tubes in three separate (x100) fields are shown in Figure 4B and C, respectively. Treatment with M867 combined to radiation significantly decreased tubule formation compared to radiation alone (5.7 vs 1.7, p<0.001). No treatment control had 13 tubules (SD = 1.0) per microscopic field and M867 alone had 9 tubules (SD = 1.0), suggesting an anti-angiogenic effect of M867 in addition to the radio-sensitization.

### Caspase 3/7 deficiency promotes radiation sensitivity by autophagy induction, in the absence of apoptosis

**Figure 5:**
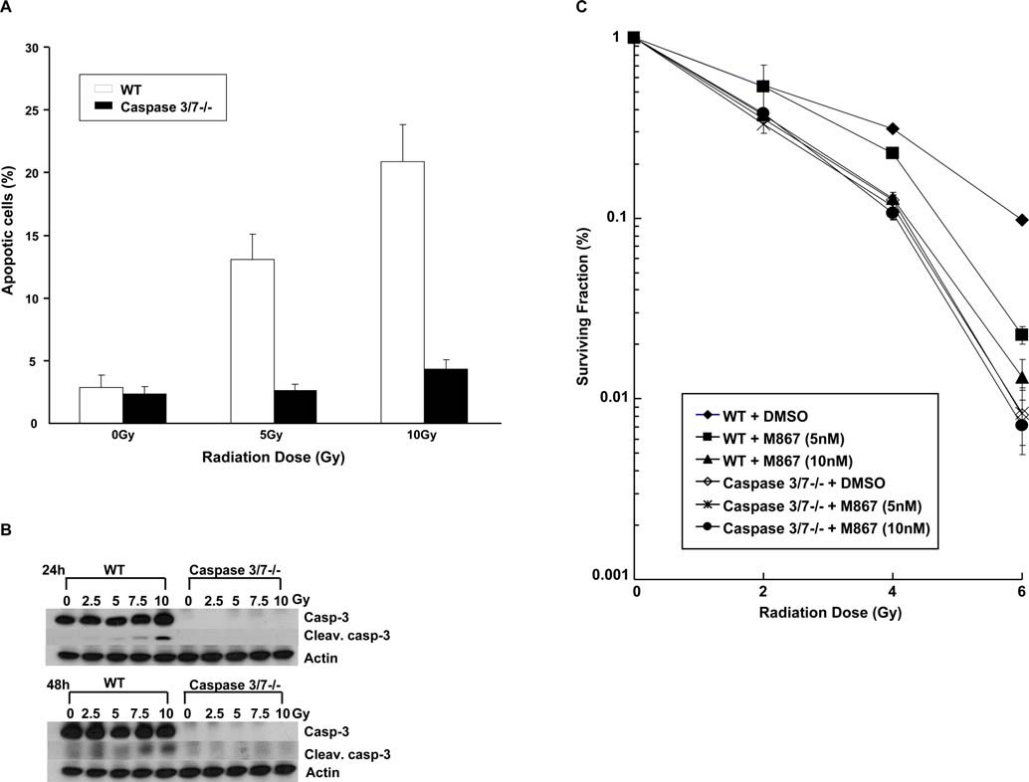
Caspase 3/7 deficiency resulted in radiation sensitivity in the absence of apoptosis in MEF cells. (A) WT and Caspase 3/7 DKO MEFs were incubated with Annexin V-fluorescein isothiocyanate and propidium iodide 24 h after irradiation and analyzed by FACScan. Data are shown as the mean of three experiments S.D. (B) Caspase cleavage was determined by immunoblotting of cleaved caspase-3 after WT or Caspase 3/7 DKO MEF cells were treated with 0, 2.5, 5, 7.5, and 10 Gy. The cells were harvested after 24. (C) Radiosensitization of WT or Caspase 3/7 DKO MEFs. The cells were irradiated with the indicated doses of radiation and treated with DMSO control or M867 (dose range from 5 to 10 nM, for 24hrs). After 10 days, colonies were stained and scored. Reported values are mean±S.D. of three separate repeated experiments.

**Figure 6:**
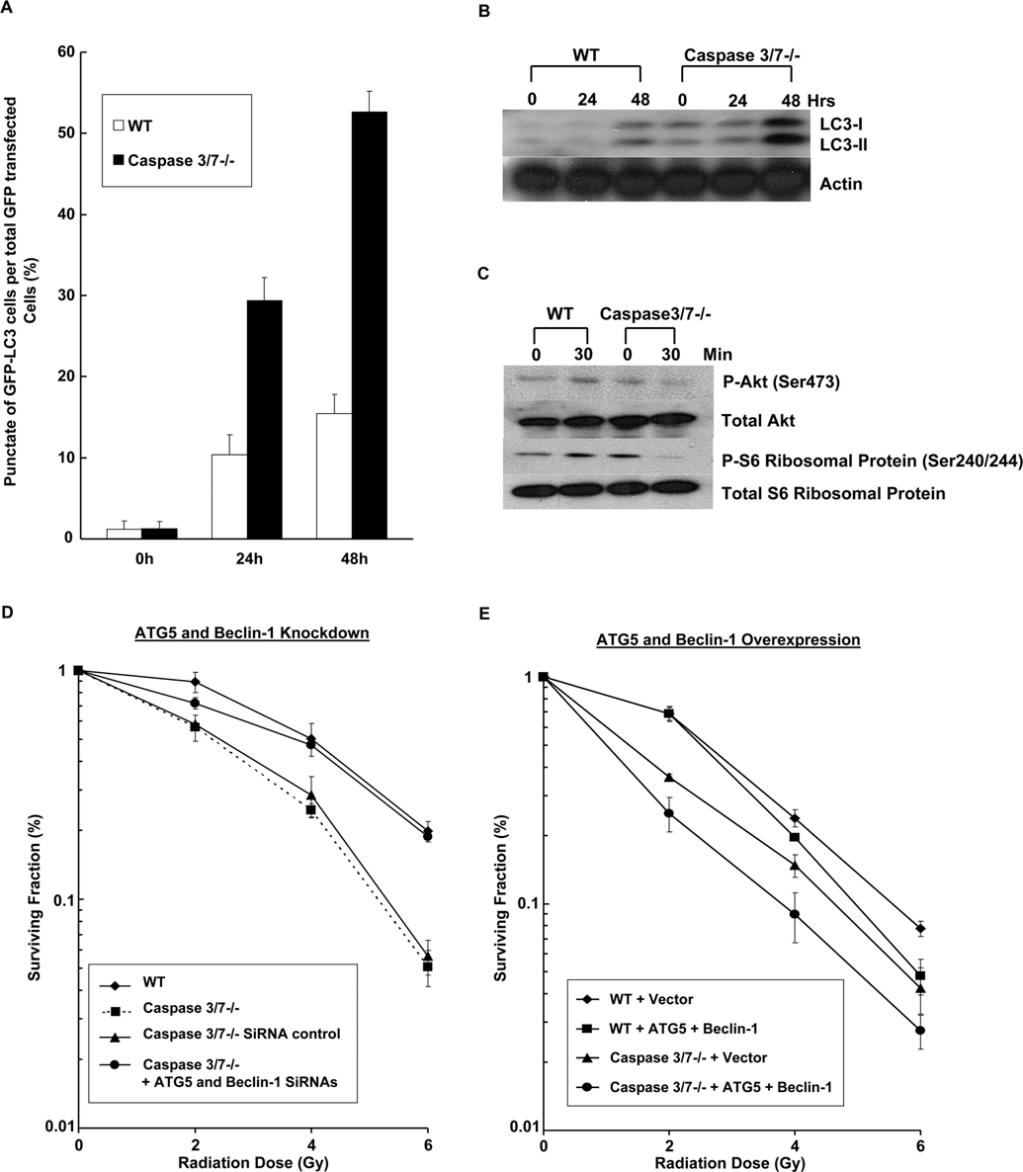
Autophagy in irradiated Caspase 3/7 DKO MEFs and effects on radiosensitivity. (A) GFP-LC3-transfected WT or Caspase 3/7 DKO MEF cells were treated with and without 5 Gy and then examined by fluorescence microscopy after 24 h. The percentage of cells with punctate GFP-LC3 fluorescence was calculated relative to all GFP-positive cells. Error bars are shown as mean S.D. (B) LC-I and –II expression was determined by Western blot using lysates from WT and Caspase 3/7 DKO MEF cells treated with 0 or 5 Gy after 24, 48 h. Actin was probed to demonstrate equal loading. (C) Also shown are immunoblots of phospho-Akt and p-S6 using the lysates from WT and Caspase 3/7 DKO MEF cells treated with 0 or 5 Gy after 30 min. (D) WT and Caspase 3/7 DKO MEF cells were transfected with either control siRNA or 25 nM siRNAs directed against ATG5 and Beclin-1 or (E) transfected with vector or ATG5 and Beclin-1 cDNA containing plasmids. They were then irradiated with 0 to 6 Gy. After 8 days, colonies were stained and scored. Values shown are the mean±S.D. of three separate repeated experiments.

To validate whether inhibition of caspases contributed to enhancing radiation therapy, caspase-3/7 deficient (DKO) MEF cells were used. These cells are unable to undergo apoptosis, since they lack caspases 3/7, whereas WT MEFs can (Figure 5A and 5B). As shown in Figure 5C, WT MEFs treated with M867 were more sensitive to radiation compared to the WT MEFs treated with DMSO (DER = 1.2 for 5nM M867, p = 0.017, and DER = 1.62 for 10NM M867, p = 0.001). There was no significant difference in survival among the caspase DKO MEFs treated with any dose of M867 as compared to DMSO. In addition, WT MEFs cells transfected with caspase-3/7 siRNAs also presented significant increase in the radiation sensitivity, suggesting that these results are not due to clonal variation of these cells (data not shown). Based on our previous study, we hypothesize that enhanced radiation sensitivity in caspase DKO cells may be due to alternative programmed cell death such as autophagy [8]. To test this hypothesis, WT and caspase 3/7 DKO cells were transfected with GFP-LC3 plasmid. Cells with punctate GFP signaling were counted as autophagic cells because of the characteristic lysosomal localization of LC3 protein during autophagy [19]. After WT and caspase DKO cells were exposed to 5Gy of radiation, the percentage of cells with GFP punctates were increased approximately 3-fold in irradiated caspase DKO cells, as compared to irradiated WT cells (30% vs 10%, respectively) (Figure 6A). At 48h post-radiation, the percentage of autophagic cells increased to ∼55% in caspase DKO cells compared to 15% in WT control. These results were also confirmed by assessing the level of processed LC3 protein, in which the caspase DKO cells showed increased levels of LC3-I and-II proteins following irradiation, in comparison to WT cells (Figure 6B). Because the mTOR pathway is known to regulate initiation of autophagy, we examined Akt/mTOR signaling by determining the levels of phospho-Akt and phospho-S6 in the irradiated WT and caspase DKO MEF cells. As shown in Figure 6C, the phospho-proteins were increased in the irradiated WT cells, whereas they were decreased in the irradiated caspase DKO MEF cells. These data suggest that pro-autophagic signaling is up-regulated in the irradiated caspase DKO cells.

### Autophagy alters radiation sensitivity in caspase 3/7 deficient cells

To determine whether autophagy is the mechanism responsible for higher radiosensitivity observed in caspase DKO cells, expression of ATG-5 and Beclin-1, two essential autophagy proteins [21], [22], [23], were knocked down in wild type and caspase DKO cells. Previous studies showed that siRNA against Beclin-1 and ATG-5 specifically downregulated endogenous protein expression [8]. As shown in Figure 6D, treatment of caspase DKO cells with siRNAs directed against ATG-5 and Beclin-1 abolished the sensitizing effect resulting from caspase-3/7 deletion and caused significant increase in clonogenic cell survival as compared to control siRNA treatment of caspase DKO cells (DER = 1.34, p = 0.008). Conversely, overexpression of Beclin-1 and ATG-5 in caspase DKO cells caused significant increase in clonogenic cell death under increasing radiation dose as compared to Beclin-1 and ATG-5 overexpression in WT cells (DER = 1.32, p = 0.008) (Figure 6E). Together, these results demonstrate that increased radiosensitivity in caspase DKO cells is dependent on key autophagy molecules.

## Discussion

In the present report, we first showed that M867 treatment resulted in the effective radiosensitization of lung cancer cells *in vitro*. The potential therapeutic effects by M867 were then demonstrated in an *in vivo* mouse hind limb tumor model. Further in vitro experiments showed that caspase-3/7-null MEF cells, were more sensitive to the cytotoxic effects of radiation, primarily due to increased autophagy. This study also suggests that M867 effects on vasculature may contribute to the observed increase in lung tumor growth delay in response to radiation.

Concurrent chemoradiotherapy is a mainstay in the treatment of advanced lung cancer, but the prognosis for those patients remains globally poor. Therefore, novel strategies are needed to improve the current treatment effectiveness by targeting of non-apoptotic cell death since apoptosis is limited following conventional therapy. Recently, there has been a great emphasis placed on autophagy as a cancer therapy target, partly driven by observations that anticancer treatments induced autophagy, such as in irradiated cancer cells [17], [24]. We showed recently that autophagy can be triggered by inhibiting the mammalian target or rapamycin (mTOR) pathway [17] or by inhibiting pro-apoptotic proteins Bak/Bax to enhance radiation effects *in vitro* [8]. A very attractive target in the apoptotic pathway is caspase-3, which has a central role in apoptosis as the dominant effector caspase involved in the proteolytic cleavage of protein substrates including cytoskeletal proteins, kinases and DNA repair enzymes. In this study, we analyzed the potential effects of caspase-3 inhibition on lung cancer response to radiation using the novel inhibitor M867. We showed that the administration of M867 in combination with ionizing radiation decreased dramatically the survival of H460 lung cancer cells (Figure 1), in a dose-dependent manner. Of note, we observed increased cytotoxicity at concentration higher than 10nM in our clonogenic assay. Nevertheless, it has been shown that M867 is a potent and reversible caspase-3 inhibitor (IC_50_ of 0.0001 μM), and a less potent caspase-7 inhibitor (IC_50_ of 0.036 μM) [18]. In addition, we found that the radiosensitizing effect of M867 is dependent upon caspase-3 and -7, as demonstrated by the absence of its impact upon caspase 3/7 DKO cells. Our study showed that increased levels of apoptosis were observed following increased doses of radiation alone in cell culture, a dose as high as 20Gy only yielded 20% of apoptotic H460 cells (Figure 1B). M867 was able to inhibit apoptosis to half at a comparable concentration (100nM) as used by Methot et al [20]. It was administered for two hours to minimize cytotoxicity. This observation is consistent with previous studies reporting the existence of defective apoptotic pathway in lung cancer. Deficient apoptosis has been long viewed as a mechanism for therapeutic resistance. Surprisingly, M867 not only reduced apoptosis following irradiation, but also to reduced tumor cell proliferation, as depicted by the Ki67 immunostaining (Figure 3A). These results can be viewed as a paradox, especially if apoptosis is the only measure of therapeutic efficacy. As we know, recent published data showed that apoptosis may account for less than 20% of radiation-induced cell death [7].

In addition, alternative death pathway such as autophagy has been demonstrated in irradiated cancer cells [17], [24]. Consistent with these results, potentiation of radiation sensitivity by vitamin D3 analogue EB 1089 has been reported in breast tumor cells via autophagy cell death [25]. In light of these data, caspase-independent cell death such as autophagy can explain the promotion of cytotoxicity in our xenograft model.

The mechanism of M867 on the radio-response in lung cancer was further characterized using a caspase-3/7 DKO model. Our results suggest the role of autophagy in the modulation of cellular sensitivity to ionizing radiation, and increased level of autophagy was determined as the possible cause of radiosensitivity when caspase 3/7 are deficient. As shown, irradiated DKO cells had elevated autophagic activity. The down-regulation or up-regulation of pro-autophagic proteins ATG5 and Beclin-1 resulted in a reduced or greater radiosensitization, respectively (Figure 6).

Additionally, our results support the potential effects of M867 on the tumor microenvironment. As shown in Figure 3A, M867 treatment alone was able to decrease Ki67 index, possibly by an effect on angiogenesis. Indeed, neovascularisation is a crucial step for tumor development, and progression of lung cancer, and high microvessel density has been identified as a prognostic factor predictive of metastasis and poor survival [26]. As shown by vFW staining (Figure 4A), the combination M867/radiation further reduced the vessels density compared to radiation alone. Our results are consistent with a report showing that blockage of caspase activity produces alterations in the normal angiogenic pattern, which suggest that active caspase may participate in angiogenesis by allowing the formation of a new functional blood vessel network [27]. As we know, cell motility and migration are critical steps of angiogenesis [28]. Interestingly, two very recent reports may bring some lights on the possible link between inhibition of caspase activity and the significant anti-angiogenic effects observed with M867. The first report showed that caspase-8 can regulate cell adhesion and motility [29]. The other study demonstrated that glioblastoma cells exhibit a basal constitutive caspase activity that is sufficient to promote migratory and invasive activities [30]. These results suggest that activation of caspases following pro-apoptotic therapy may enhance metastasis or invasiveness of apoptosis-resistant cancer cells. This would lead us to speculate that instead of inducing cancer cytotoxicity, such treatment may unexpectedly promote tumor migration and metastasis. Therefore, M867 may serve to suppress these unwanted consequences in irradiated cancer cells which survived apoptotic cell death. We also examined the effect of M867 on the angiogenic function of HUVECs *in vitro*, using an endothelial cell morphogenesis assay (Figure 4, B and C). The bi-modality treatment decreased the ability of endothelial cells to form capillary tubule-like structures compared with M867 or radiation alone. One of the molecular mechanisms for radioresistance is the production of pro-angiogenic factors such as the vascular endothelial growth factor (VEGF), fibroblast growth factor (FGF) and platelet-derived growth factor (PDGF) following irradiation [31], [32], [33], [34]. Radiation-induced activation of angiogenesis thus attenuates vasculature damage under radiotherapy and potentially limits the effectiveness of this anticancer modality. As a fundamental step in tumorigenesis and in radioresistance, angiogenesis represents an attractive therapeutic target, and this report shows that M867 provides an effective means of inhibiting neovascularisation in irradiated lung tumor models.

The stimulation of autophagy was shown to enhance radiation cytotoxicity in cancer cells [35], [36]. A similar effect on vasculature was supported by a report from Chau et al that showed autophagic cell death also inhibits angiogenesis [37]. PTEN, a tumor suppressor protein frequently inactivated in cancer, is associated with autophagy, and was found to suppress tumor neovascularization in an in vivo brain tumor model [38]. Loss of PTEN leads to the enhancement of tumor angiogenesis in mouse endothelial cells [36]. These data support our conclusion that cancer therapy can be enhanced by promotion of autophagy within both cancer cells and the vasculature.

A major limitation in delivering thoracic radiotherapy for lung cancer is to achieve an optimal radiation dose for maximum tumoricidal effect without injuring the normal lung tissue. Radiation pneumonitis, an interstitial pulmonary inflammation that develops in up to 30% of patients after thoracic irradiation [39], and subsequent interstitial lung fibrosis [40] are challenging dose-limiting toxicities of radiation therapy for lung cancer. Thus, the development of a strategy that could protect lung from radiation pneumonitis and fibrosis would offer the possibility of improving the therapeutic ratio of patients with lung cancer. Several caspases have been associated with inflammatory diseases and mediation of pro-inflammatory cytokines [41]. Moreover, there is increasing evidence that apoptosis might play a role in several pulmonary diseases [42], including fibrosing pulmonary diseases, and bronchiolitis obliterans-organizing pneumonia [40]. Fas/FasL-mediated lung epithelial apoptosis can lead to inflammation and progression from ARDS to fibrosis [42]. A role of TGF-beta 1 in the pathophysiology of pulmonary fibrosis as an enhancer of Fas-mediated apoptosis of lung epithelial cells and lung injury via caspase-3 activation was also previously demonstrated in mice [43]. It is also well known that irradiation of the lungs results in an increased expression of TGF-beta which has been reported to be related to the development of radiation-induced pneumonitis and fibrosis [44]. One could thus hypothesize that the inhibition of caspase may protect healthy surrounding lung tissues against thoracic radiation injury. This hypothesis is also based on the high apoptosis level found in irradiated normal lung cells as compared to the lung cancer cells which are resistant to apoptosis. This possible radio-protective role of caspase inhibition deserve further pre-clinical study using, for example, C57BL/6 mice [45], [46]. More precisely, C57BL/6 mice that express wild-type levels of ICAM-1 show pulmonary inflammation following thoracic irradiation beginning within 4 weeks of irradiation, whereas C57BL/6 mice that lack ICAM-1 gene expression do not develop inflammation.

This preclinical study not only supports the significant therapeutic potential of M867 as a radiosensitizer in lung cancer but also suggest caspase inhibition as a novel concept for the enhancement of radiation therapy in cancer. This promising strategy needs further specific radiotherapy-driven experiments and clinically relevant study to explore novel combinations using this proof-of-principle.
